# Microbial Enzymes and Their Applications in Industries and Medicine

**DOI:** 10.1155/2013/204014

**Published:** 2013-12-02

**Authors:** Periasamy Anbu, Subash C. B. Gopinath, Arzu Coleri Cihan, Bidur Prasad Chaulagain

**Affiliations:** ^1^Department of Biological Engineering, Inha University, Incheon 402-751, Republic of Korea; ^2^Nanoelectronics Research Institute, National Institute of Advanced Industrial Science and Technology, 1-1 Higashi, Tsukuba, Ibaraki 305-8562, Japan; ^3^Department of Biology, Faculty of Science, Ankara University, Tandogan, Ankara 06100, Turkey; ^4^Department of Plant Biology and Biotechnology, University of Copenhagen, Copenhagen, Denmark

Enzymes are considered as a potential biocatalyst for a large number of reactions. Particularly, the microbial enzymes have widespread uses in industries and medicine. The microbial enzymes are also more active and stable than plant and animal enzymes. In addition, the microorganisms represent an alternative source of enzymes because they can be cultured in large quantities in a short time by fermentation and owing to their biochemical diversity and susceptibility to gene manipulation. Industries are looking for new microbial strains in order to produce different enzymes to fulfil the current enzyme requirements. This special issue covers ten articles including three review articles, mainly highlighting the importance and applications of biotechnologically and industrially valuable microbial enzymes.

M. Dinarvand et al. in their paper optimized the conditions for overproduction of intraextracellular inulinase and invertase from the fungus *Aspergillus niger* ATCC 20611. Optimization is one of the most important criteria in developing any new microbial process. Response surface analysis is one of the vital tools to determine the optimal process conditions. This kind of design of a limited set of variables is advantageous compared to the conventional method. The response surface methodology was used for this optimization and achieved the increment until 16 times. This study would be highly useful for the potential application in fermentation industries.

In this review, N. Gurung et al. have made an attempt to highlight the importance of different enzymes with a special focus on amylase and lipase. Enzymes generally increase the reaction rates by several million times than normal chemical reactions. Lipases play an important role in the food, detergent, chemical, and pharmaceutical industries. In the past, microbial lipases gained significant attention in the industries due to their substrate specificity and stability under varied conditions. Amylase is an enzyme that catalyses the breakdown of starch into sugars, abundant in the process of animal and human digestion. The major advantage of microbial amylases is being economical and easy to manipulate. Currently, much attention is paid to rapid development of microbial enzyme technology, and these enzymes are relatively more stable than the enzymes derived from plants and animals.

P. Mukherjee and P. Roy in their paper have purified and characterized the enzyme hydrocarbon dioxygenase from *Stenotrophomonas maltophilia *PM102, which has a broad substrate specificity. They found that the presence of copper induces the enzyme activity to be 10.3-fold higher, and NADH induces the increment to be 14.96-fold. Proposed copper enhanced monooxygenase activity and Fourier transform-infrared (FT-IR) characterization of biotransformation products from trichloroethylene satisfy the production of industrially and medically important chemicals and make bioremediation more attractive by improving the development of this technology.

C. Huynen et al. in their review paper discuss the importance of protein scaffold to develop hybrid enzymes. The paper discusses the use of class A betalactamase as versatile scaffolds to design hybrid enzymes mentioned as betalactamase hybrid proteins (BHPs), in which an external polypeptide, peptide, protein, or their fragment is inserted at various suitable positions. The paper highlights further how BHPs can be specifically designed to develop as bifunctional proteins to produce and characterize the proteins otherwise difficult to express, to determine the epitope of specific antibodies, to generate antibodies against nonimmunogenic epitopes, and to understand the structure/function relationship of proteins. The hybrid proteins can be applied to produce difficult-to-express peptides/proteins/protein fragments, to map epitopes, to display antigens, and to study protein structure/function relationships. Among other applications, BHPs could be an important player in biosensors and in affinity chromatography, drug screening, and drug targeting.

P. Manivasagan et al. in their paper focus on purification and characterization of the protease from *Streptomyces* sp. MAB18. The authors have optimized the conditions for overproduction of protease using response surface methodology. They have also determined the molecular mass of purified enzyme and great activity and stability of enzyme in different pH and temperatures. Furthermore, the authors confirmed that the protease has an antioxidant ability. In industries, the poultry waste derived protease will be useful as a protein or as an antioxidant.

The paper titled “**β*-Glucosidases from the fungus Trichoderma: an effeicient cellulose machinery in biotechnological applications*” is a detailed review on **β**-glucosidases which are members of the cellulose enzyme complex described by P. Tiwari et al. The authors especially focus on **β**-glucosidases from the fungus *Trichoderma,* mostly used for the saccharification of cellulosic biomass for biofuel production. They describe the enzyme family, their classification, structural parameters, properties, and studies at the genomics and proteomics levels. In addition, by bypassing the low enzyme production with hypersecretory strains, they give an insight on using these strains for renewable energy sources like bioethanol production. They imply the importance of fungal **β**-glucosidases which might be successful for biofuel production in order to meet the need in energy crisis.

A. Khoramnia et al. in their paper discuss yeast enzyme application for medium chain fatty acids (MCFAs) modification for industrial purpose and antibacterial applications. The paper focuses on the conceptualization, design, and assay of the enzyme produced from a Malaysian strain of *Geotrichum candidum*. With the modification on fatty acid processing using a naturally derived enzyme, a free lauric acid rich MCFAs can be obtained which can become a source of antibacterial use for both Gram-positive (*Staphylococcus aureus*) and Gram-negative (*E. coli*) bacteria which are difficult microbes due to some of their strains becoming drug resistant. They also describe that the higher lipolysis by the strain specific enzyme is associated with the increased moisture content in the reaction environment on coconut oil hydrolysis.

M. A. Hassan et al. in their paper discuss isolation of *Bacillus amyloliquefaciencs* and *B. subtilis* from soil and production and characterization of keratinolytic protease. These bacteria were able to degrade the wool completely within 5 days and also produced the highest enzyme activity. The characterization studies confirmed that the enzyme is stable in a broad range of pH and temperatures. Furthermore, they confirmed that the keratinolytic proteases from isolated bacteria are stable in various organic solvents.

In this review article, S. C. B. Gopinath et al. put different strategies to characterize fungal lipases for their role in industry and medicine. The advantage of fungal lipases is bestowed with their extracellular nature of production thus reducing the complexities and high operation cost comparing to other bacterial enzymes. The authors provide several illustrations to show how lipolysis can be utilized and put strategies for the characterization of fungal lipases that are capable of degrading fatty substances from different sources, with an effort to highlight further applications. This review would contribute to the isolation and characterization of lipase from various fungal sources and application of lipase for medical and dairy industry and degradation of fatty substance from oil spillages.

A. Knob et al. in their paper focus on xylanses and discuss the purification and characterization of a xylanase produced by *Penicillium glabrum* using brewer's spent grain as a substrate in their paper. This study is the first report as the characterization of xylanase was carried out by using such an agroindustrial waste. Furthermore, the researchers also determined the molecular mass of the purified xylanase, the enzyme activity and stability on various pH and temperature ranges, the optimal enzyme production conditions, and the effect of some metal ions and inhibitors on xylanase activity. The authors concluded that the use of substrate brewer's spent grain for xylanase production not only decreased the amount of this waste but also reduced the xylanase production cost as desired in biotechnological processes.

*Periasamy Anbu* *Subash C. B. Gopinath* *Arzu Coleri Cihan* *Bidur Prasad Chaulagain*

